# The Effect of Adenotonsillectomy on Children’s Behavior and Cognitive Performance with Obstructive Sleep Apnea Syndrome: State of the Art

**DOI:** 10.3390/children8100921

**Published:** 2021-10-15

**Authors:** Paola Di Mauro, Salvatore Cocuzza, Antonino Maniaci, Salvatore Ferlito, Deborak Rasà, Roberta Anzivino, Claudio Vicini, Giannicola Iannella, Ignazio La Mantia

**Affiliations:** 1 Department of Medical and Surgical Sciences and Advanced Technologies “G.F. Ingrassia”, ENT Section, A.O.U. “Policlinico—Vittorio Emanuele”, University of Catania, 95124 Catania, Italy; s.cocuzza@unict.it (S.C.); tnmaniaci29@gmail.com (A.M.); ferlito@unict.it (S.F.); deborak.rasa@hotmail.it (D.R.); igolama@gmail.com (I.L.M.); 2 Otolaryngology Unit, Di Venere Hospital, 70131 Bari, Italy; roberta.anzivino@gmail.com; 3 Head-Neck and Oral Surgery Unit, Department of Head-Neck Surgery, Otolaryngology, Morgagni Piertoni Hospital, 47121 Forlì, Italy; claudio@claudiovicini.com; 4 Organi di Senso Department, Sapienza University, 00185 Rome, Italy; giannicola.iannella@uniroma1.it

**Keywords:** sleep disordered breathing, obstructive sleep apnea syndrome, pediatric sleep apnea, pediatric behavior disorders, adenotonsillectomy

## Abstract

(1) Background: This systematic review was designed to analyze adenotonsillectomy’s role in treating behavioural disorders and sleep-related quality of life in pediatric OSAS. (2) Methods: Papers that report pre-operative and post-operative outcomes by using the Epworth sleepiness scale, OSA-18, NEPSY, Conners’ rating scale, BRIEF, PSQ-SRBD, PedsQL and CBCL. We performed a comprehensive review of English papers published during the last 20 years regarding behavioural disorders in OSAS patients and adenotonsillectomy. (3) Results: We included 11 studies reporting behavioral outcomes and sleep related quality of life after surgery. We investigated changes in behavior and cognitive outcomes after AT, and we found significant improvements of the scores post-AT in almost all studies. After comparing the AT group and control group, only one study had no difference that reached significance at one year post-AT. In another study, it did not show any significant improvement in terms of all behavioural and cognitive outcomes. The questionnaires on sleep-related quality of life after AT (PSQ-SRBD or ESS or OSA-18 or KOSA) may improve with positive changes in sleep parameters (AHI, ODI and SpO2). Furthermore, there is a significantly higher decrease in OSAS symptoms than the pre-AT baseline score. (4) Conclusion: Future studies should pay more attention to characterizing patient populations as well as rapid surgical treatments through existing criteria.

## 1. Introduction

Obstructive Sleep Apnea Syndrome (OSAS) is a condition characterized by repeated episodes of complete (apnea) or partial (hypopnea) cessation of airflow through the upper airways during sleep [1,2]. The prevalence of respiratory sleep disorders in preschool and school-age varies widely, with an estimated rate of primary snoring in children ranging from 8% to 27% and of OSAS from 1% to 5% [3,4,5,6,7,8].

The OSAS symptomatology in pediatric age is usually unclear and is characterized by poor school performance, daytime sleepiness, growth deficit and nocturnal enuresis [9,10,11,12,13,14,15,16,17,18,19,20,21,22,23,24]. Moreover, children with OSAS present behavioral disorders such as irritability, attention deficit, sleepiness or hyperactivity in contrast, emotional lability and aggressiveness [13,25,26,27,28,29,30,31,32,33,34]. Neurocognitive deficits and delayed growth are related to hypoxemia micro awakenings and sleep fragmentation [35,36,37,38,39]. 

Kennedy et al. in 2004 reported impaired memory, global intelligence and selective attention in patients with ≥3% oxygen desaturation in REM sleep [35]. Moreover, primary snoring has been associated with neurocognitive disorders [15,36,40]. Over time, there have been numerous mostly cross-sectional studies reporting the association between OSAS and neurocognitive and behavioral morbidity [41,42,43,44,45,46]. 

Brockmann et al. in 2011 analyzed a sample of 1114 school children, reporting significant differences between “never snoring”, PS (Primary Snoring) and UARS/OSA (Upper Airway Resistance Syndrome/Obstructive Sleep Apnea) (*p* < 0.0001) in both hyperactive and inattentive behavior scores. These characteristics were more frequently reported in children with PS compared to “never snorers” (OR > 1) (Cohen’s d = −0.75 (medium effect size)) [15].

Palatine tonsil hypertrophy is the most common cause of upper airway lumen reduction in children [10] that is frequently associated in preschool children and frequently associated with obesity [4,20]. 

Many papers in the literature have addressed the same topic in adults. It is worth noting that OSAS risk factors are somewhat different. In adults, the risk factors involved include anatomy and function of the upper airways; control of ventilation; familiarity; sex; snoring; endocrine-metabolic diseases; arterial hypertension; gastro-esophageal reflux; epilepsy; tobacco and alcohol abuse; anesthetic and tranquilizers drugs; caffeine; and menopause in the woman [47].

Usually, snoring in children is considered “benign”, especially in the absence of significant obstructive events. Contrariwise, hypoxemia commonly involving OSAS disease can be associated with micro awakenings, fragmentation of sleep and is related to daytime symptoms [12].

In the adult population, OSAS results in daily sleepiness, daytime fatigue, systemic hypertension, abnormality in regularity of heart rate fluctuations and affects cognitive function too. Hypertension and cardiovascular risks are not the only OSAS consequences; depression, cognitive impairment and neurodegenerative disorders are really important too [48,49]. Psychiatric comorbidities, especially depressive disorders, are often described in adults with OSAS because the disturbed sleep pattern negatively affects the stress system and increases the susceptibility of OSAS patients to depression [49].

Nevertheless, the impact of OSAS in children’s cognitive functions is steadier, and it affects the correct psychophysical growth of children [49,50]. 

While the gold standard to treat OSAS is the Continuous Positive Airway Pressure (CPAP) method in the adult population, in children, AT remains as OSAS’s first therapeutic choice, especially in cases of moderate to severe apnea (AHI > 5) [1,34,51]. However, the efficacy of adenotonsillectomy (AT) is still debated.

Wei et al. found improvements in both sleep and behavior in 6 months outcomes after AT for OSAS [46]. At follow-up T scores on CPRS-RS (Conners’ Parent Rating Scale–Revised Short Form) index for cognitive impairment (Cohen’s d = 0.725 (medium effect size)), oppositional behavior (Cohen’s d = 0.71 (medium effect size)), hyperactivity (Cohen’s d = 0.758 (medium effect size)) and ADHD (Cohen’s d = 0.848 (large effect size)) were statistically significant (*p* < 0.001) [46].

On the other hand, Landau et al. have found how behavioral and neurocognitive functions of children with OSAS were impaired compared to healthy children; indeed, in their study, the quality of life questionnaire in children with OSAS was significantly worse compared to controls (*p* < 0.004) (Cohen’s d = 0.558 (medium effect size)). One year after AT, the following neurobehavioral functions were significantly improved (*p* < 0.05), and differences in these functions between children with OSAS after TA and healthy children were not observed [42]. 

In confirmation of possible chronic psychophysiological stress, in their meta-analyses and meta-regressions about the comparison between children with OSAS and healthy controls, Imani and colleagues demonstrated that plasma levels of IL-6 ((95% CI: 0.27, 1.41; *p* = 0.004; I^2^ = 72% (P_h_ = 0.03))) [52] and serum levels of TNF-α (0.21 pg/mL (95% CI = 0.05, 0.37; *p* = 0.01), I^2^ = 77% (P_h_ = 0.005)) [53] were significantly higher in children with OSAS, while the morning saliva cortisol concentrations were significantly lower (MD = −0.13 µg/dL; 95% CI: 0.21, −0.04; *p* = 0.003 I^2^ = 0% (P_h_ = 0.34)) in children with OSAS [54].

On the other hand, in a retrospective study, Gozal et al. analyzed 797 subjects with low performance (LP) and 791 subjects with high performance (HP) among seventh and eighth graders attending public schools by using questionnaires. The authors reported snoring in early childhood in 103 LP children versus 40 in HP children (5.1%; OR: 2.79; confidence interval (CI): 1.88–4.15; *p*, 0.00001), with AT surgical intervention in 24 LP and 7 HP children (odds ratio: 3.40; confidence interval: 1.47–7.84). These data suggested that neurocognitive morbidity may only be partially reversible after treatment and that residual deficits in the learning process could still remain many years after snoring has resolved. [33].

Moreover, Kohler and colleagues assessed by means of the Stanford Binet Intelligence Scale 5th edition, Neuropsychological Developmental Assessment (NEPSY) and polysomnography a total of 44 healthy snoring children (aged 3–12 years) at baseline and 6 months after adenotonsillectomy and reported the comparison with 48 age and gender matched non-snoring controls. In this case, neurocognitive deficits were reported at baseline in snoring children when compared to controls (10 point IQ difference, with *p* = 0.001, Cohen’s d = 0.929 (large effect size) for full scale IQ); however, neurocognitive deficits did not improve 6 months after surgery relative to controls (Cohen’s d = 0.137 (trivial effect size), although the range in frequency of desaturation was extremely reduced (from 0–53.1 to 0–5.6) [24]. 

In light of such heterogeneous results, it seemed reasonable to analyze literature data of the last 20 years on pediatric OSAS patients and provide a systematic review about the current correlation between AT and neurocognitive/behavioral disorders. 

In particular, we examined the efficacy of AT on behavior through the use of validated questionnaires on sleep parameters compared from baseline to follow up after AT and, whenever possible, to subjects not treated surgically with AT (WWSC (Watchful Waiting with Supportive Care group) or control group).

## 2. Materials and Methods

### 2.1. Protocol Data Extraction

**Figure 1:**
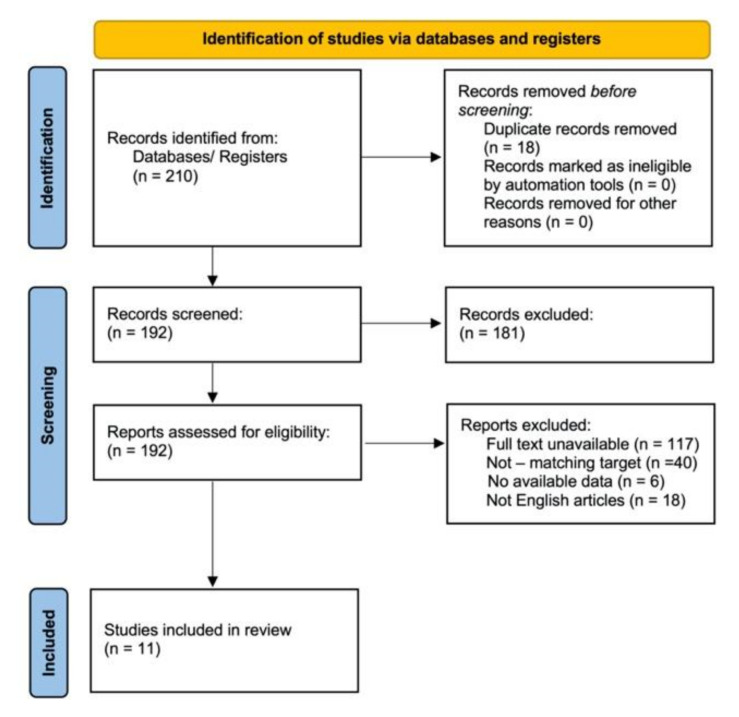
PRISMA flow diagram.

According to the PRISMA checklist for review and meta-analysis, we performed a systematic review of the current literature [55] (Figure 1), and this review protocol was registered on the International Prospective Register of Systematic Reviews (PROSPERO; registration number: 277325).

The authors P.DM and I. LM searched the Medline database via PubMed, EMBASE and Cochrane library from January 2001 to April 2021, solving any disagreements among the study members through a discussion. 

We examined all the studies included, analyzing all available data and guaranteeing eligibility for all subjects. Main patient features, symptoms, diagnostic procedures, treatment modalities, outcomes scores and follow-up were collected. In order to analyze sleep quality, we analyzed data from AHI (Apnea Hypopnea index), ODI (Oxygen Desaturation Index), OSA- 18 items, PSQ-SRBD (Sleep-Related Breathing Disorder scale of the Pediatric Sleep Questionnaire), mESS (Epworth Sleepiness Scale modified for children), SpO2, KOSA-18 (Korean version of the obstructive sleep apnea-18), pediatric daytime sleepiness scale and mean sleep latency. 

In order to collect data about behavioral disorders, we analyzed data from NEPSY (Developmental Neuropsychological Assessment); NEPSY-II (Developmental Neuropsychological Assessment II edition); CRS-R (Conners’ Rating Scale-Revised); CTRS (CTRS = Conners’ Teacher Rating Scale); BRIEF (Behavior Rating Inventory of Executive Function); PedsQL (Pediatric Quality of Life Inventory); DAS-II (Differential Abilities Scales, 2nd edition); Purdue Pegboard Test; Developmental Test of Visual-Motor Integration; WRAML2 (Wide Range Assessment of Memory and Learning, 2nd edition); CBCL (Child Behavior Checklist); DST (Digit Span Test); COWAT (Controlled Oral Word Association Test); TOL (Tower of London); RCPM (Raven’s Colored Progressive Matrices); K-ARS (Korean ADHD rating scale); Children’s Global Assessment Scale CGI (Clinical Global Impressions); Cognitive Attention Index Behavioral hyperactivity index; and ADHD rating scale. 

### 2.2. Electronic Database Search

PubMed/Medline, Embase, Web of Science, Scholar and the Cochrane Library electronic databases were searched for studies on adenotonsillectomy in OSA pediatric patients and neurocognitive and behavioral disorders over the last 20 years of literature (from 1 July 2001 to 1 July 2021) by two different authors. We used the following search keywords: “OSAS”, “Obstructive Sleep Apnea Syndrome”, “Sleep-Disordered Breathing”, ‘’adenotonsillectomy”, “cognitive disorders,’’ “behavior”, “neurocognitive function” and “quality of life”.

All the papers’ titles and abstracts available in the English language were analyzed; thus, we identified full-text articles screened for original data. The search process is summarized in Figure 1.

### 2.3. Inclusion Criteria 

Studies that met the following criteria were included:

(1) Cross-sectional studies, case controls, retrospective cohort studies, prospective cohort studies, primary science articles and epidemiological studies;
(2) Studies regarding children with OSAS treated with adenotonsillectomy;
(3) Studies using at least one validated questionnaire on the behavior of children with OSAS before and after adenotonsillectomy.
(4) All the studies reported detailed information on preoperative and postoperative OSA cognitive, behavioral and/or sleep outcomes, such as AHI, ODI, CRS-R, CBCL, NESPI and BRIEF.

### 2.4. Exclusion Criteria Selected

(1) Articles not published in English;
(2) Case reports, letters to the editor and reviews;
(3) Papers missing preoperative and postoperative continuous data.

The process undertaken is schematically presented in Figure 1.

For each study, we reported the following clinical characteristics: type of behavior and/or sleep questionnaires; correlation of the results of the questionnaires before and after tonsillectomy.

## 3. Results

### 3.1. Retrieving Research

According to the PRISMA checklist for review and meta-analysis, we reviewed 210 articles. Before screening, 18 of them were removed because they were duplicate records, and 192 were assessed for eligibility. Of these, however, 117 were removed due to the full-text being unavailable, 40 were removed because they did not analyze our search target, 6 were removed because data were not available and 18 were removed because they were not written in English. 

At least eleven papers (2776 patients) were considered eligible for our analysis [3,56,57,58,59,60,61,62,63,64,65], of which four were prospective cohort studies [58,61,62,64] and seven were randomized controlled trials [3,56,57,59,60,63,65] (see Table 1).

The main features of the data of the included articles and the studies measured with time of follow-up are summarized in Table 2.

We found an age range from 5.0 to 12.9 years old. Six studies [3,56,57,59,62,63] reported the number of overweight or obese children, that is, 1069/2712 (39,41%). In particular, 780 (28.76%) overweight or obese children received AT. 

Most of patients performed polysomnography, reporting Apnea/Hypopnea Index (AHI) [3,56,57,58,59,60,61,63,64,65], Oxygen Desaturation Index (ODI) [52,54,56,60] or Obstructive Apnea Index (OAI) [61]. Some studies further measured percentage sleep time with end-tidal CO2 values > 50 mmHG [55,56], OSA-18 items [56,60,64] or the Korean version of it (KOSA-18) [63]. Quality of life was measured by using the Pediatric Quality of Life (PedsQL) [3,60]. To evaluate neuropsychological assessment, neuropsychological test batteries such as NEPSY were used [3,56,59,60]. 

### 3.2. Patient Features and Surgery

In particular, we provided 11 articles with a total of 2712 patients [3,56,57,58,59,60,61,62,63,64,65]. In particular, 1455/2712 (53.65%) patients received AT, while 1061/2712 (39.12%) patients were assigned to watchful waiting with supportive care (WWSC) group. The WWSC group was present in six papers [3,56,57,59,64,65].

All studies analyzed the efficacy of AT on cognitive or behavioral measures outcomes assessed by validated questionnaires. The main results of Baseline Outcomes in WWSC or control and AT groups are summarized in Table 3. The main outcomes at follow-up and change from baseline to follow-up between groups are summarized in Table 4.

### 3.3. Neurocognitive Performance

Four articles, three RCTs [3,56,59] and one prospective cohort studio [58] evaluated the effect of AT on the results of the neurocognitive performance (Table 4).

In Marcus et al.’s study, the average on the NEPSY scores in comparison between early adenotonsillectomy group and WWSC group showed a difference but was not significant (*p*-value = 0.16) (Cohen’s d = 0.15 (small effect size)) [3]. 

In Taylor et al.’s study, AT confers small positive effects on cognitive test scores in children with OSAS without prolonged desaturation and with overall average cognitive functioning. Tests of nonverbal reasoning, attention and fine motor skills were found selectively affected by OSAS and improved after AT (Cohen’s d = 0.20–0.24 (medium effect size)). However, Neuropsychological Test Battery (Purdue Pegboard Non-dominant (β(SE) = −0.06 (0.11), *p* = 0.580) or Both Hands (β(SE) = 0.18 (0.08), *p* = 0.031), NEPSY Visual Attention (β(SE) = 0.6 (0.32), *p* = 0.061), DAS-II Pattern Construction (β(SE) = −0.76 (0.62), *p* = 0.223), NEPSY Auditory Attention and Response Set (β(SE) = 0.21 (0.23), *p* = 0.353), NEPSY-II Inhibition Naming Condition (β(SE) = 0.13 (0.40), *p* = 0.739), NEPSY-II Word, Generation Semantic Condition (β(SE) = 0.07 (0.27), *p* = 0.797) and Wide Range Assessment of Memory and Learning, 2nd edition ((WRAML2) Verbal Learning) (β(SE) = −0.02 (0.27), *p* = 0.935) at baseline and follow-up have not noted group differences significant at comparisons with the control group [56].

Khalid Al-Zaabi et al. in the AT group showed significant improvements in all neurocognitive function parameters including attention/concentration (42%), (Cohen’s d = −0.773 (trivial effect size)), executive function (52%) (Cohen’s d = −1.201 (trivial effect size)), learning/recall (38%) (Cohen’s d = −1.249 (trivial effect size)), verbal fluency (92%) (Cohen’s d = −0.792 (trivial effect size)) and general intellectual ability (33%) (Cohen’s d = −0.81 (trivial effect size)) (*p*-value < 0.01) [58]. 

Shalini Paruthi et al. analyzed the correlation between Hypercapnia and Cognitive Outcomes. The baseline percentage of Total Sleep Time (TST) with EtCO2 > 50 mmHg did not correlate with changes on the cognitive assessments at follow-up (r = 0.09 to 0.012, all *p* > 0.15) even after adjustments for age, sex, race and the treatment assignment (*p*-value > 0.3) [59].

### 3.4. Behavioral Outcomes

Ten articles, six RCT [3,57,59,60,63,65] and four prospective cohort studies [58,61,62,64] evaluated the effect of AT on the results of the behavioral assessment (Table 4).

Marcus et al. reported a significant improvements among early adenotonsillectomy group than among WWSC group in behavioral disorders assessed via the caregiver-reported Conners’ Rating Scale, the teacher-reported data and the caregiver-reported BRIEF. However, they were not significantly different in terms of the teacher-reported version between the groups (*p*-value = 0.04) (Cohen’s d = 0.29 (medium effect size)) [3].

In Hattiangadi Thomas et al.’s paper, at follow-up, Total Problems (change from baseline to 7 months: −1 (−6, 4), (*p* < 0.001)) and Internalizing (change from baseline to 7 mounths: −1 (−6, 6), (*p* = 0.04)) were evaluated. CBCL T-scores reduced more in eAT than WWSC. The eAT group also proved a significantly greater decrease in Thought Problems and Somatic Complaints [57].

In Al-Zaabi et al.’s study, a significant reduction of 21% in both ADHD inattention (Cohen’s d = 0.916 (large effect size)) and hyperactivity scores (Cohen’s d = 0.732 (medium effect size)) (*p* < 0.01 each) was noted; however, the mean post-AT hyperactivity score, nevertheless, remained above a cut-off value of >15 (15.84 ± 4.13) [58].

In Paruthi et al.’s study, Hypercapnia and Behavioral Outcomes were correlated. The baseline percentage of TST with EtCO2 > 50 mmHg did not correlate with changes on behavioral assessments at follow-up (all *p*-value > 0.05), even after adjustments for age, sex, race and the treatment assignment (*p* > 0.3, Spearman: −0.059) [59].

Dillon et al. reported that the frequency of psychiatric disorders among controls changed minimally from baseline to follow-up (Cohen’s d = 0.161 (trivial effect size)).

In contrast, the frequency of attention and disruptive behavior disorders in AT children dropped from 36.7% to 23.1% (*p* = 0.008) (Cohen’s d = 0.359 (small effect size)). The overall prevalence of ADHD declined modestly from 27.8% to 20.5% (Cohen’s d = 0.246 (small effect size)). ADHD remitted after AT (among 11 patients); rating changes on the DBDRS subscales corresponding to pre-operative diagnosis changed by an average of 51.1% [61].

In Jeon et al.’s study, the mean Korean ADHD rating scale (K-ARS) score at preoperative 1 day was 12.5 ± 9.7, which improved to 7.0 ± 6.4 at postoperative 1 month (*p*< 0.01) and was still significantly lower than the preoperative at the 6-month follow-up with a score of 8.4 ± 7.7 (*p* < 0.01). In particular, the mean preoperative attention deficit and hyperactivity-impulsivity domain scores (6.2 ± 5.3 and 6.2 ± 5.1, respectively) reduced significantly at postoperative 1 month (3.1 ± 3.2 (Cohen’s d = 0.708 (medium effect size)) and 3.9 ± 3.6 (Cohen’s d = 0.521 (medium effect size)), respectively, and at 6 months (4.1 ± 3.8 (Cohen’s d = 0.455 (small effect size)) and 4.7 ± 4.7 (Cohen’s d = 0.306 (small effect size)), respectively, (all *p*< 0.01) [63].

In Rosen et al.’s study, the behavioral health measures (NEPSY-A/E score (Cohen’s d = −0.43 (medium effect size)), BRIEF score (Cohen’s d = 0.30 (medium effect size)), Conners’ Rating Scale (Cohen’s d = 0.26 (medium effect size)) and CBCL score (Cohen’s d = 0.34 (medium effect size)) improved significantly after adenotonsillectomy (all *p*-value < 0.001). In contrast, from their logistic regression, no behavioral, sleepiness or quality of life outcomes were predicted independently by baseline AHI [60].

The mean Pediatric Quality of Life (PedsQL), in which scores range from 0 to 100 and higher scores indicate better quality of life changing from baseline to 7 months, was −0.3 ± 0.2. In particular, 80% of the PedsQL total scores were < 0.33, suggesting high risk for obstructive sleep apnea. PedsQL scores were significantly related with AHI and the ODI at both baseline and follow-up but were not related with higher Etco2 values or other PSG measures. PedsQL (Child) and PedsQL (Parent) improved significantly after adenotonsillectomy (*p*-value: 0.006, (Cohen’s d = −0.23 (medium effect size)) and <0.001 (Cohen’s d = −0.37 (medium effect size)), respectively.

In Chervin et al., there were great differences in the behavioral hyperactivity index (*p*-value 0.003) and cognitive attention index (*p*-value = 0.020) at baseline compared to AT group and control group. In contrast, none of these differences reached significance at one year (*p*-value = 0.056 and *p*-value = 0.133, respectively). The frequency of Attention-Deficit/Hyperactivity Disorder was not significant, and the rates were not different from baselines after AT in both groups enrolled (Fisher’s Exact Test, *p*-value = 0.23) [62].

In Chun T. Au et al., patients of both AT and WW groups had fewer behavioral problems at follow-up as reported by their parents on the CBCL compared to baseline (*p* < 0.05, Cohen’s d = 0.15 (small effect size)). 

However, there were no significant between-group differences in the changes of any CBCL score, and there were no significant differences in the changes in CPT parameters, CBCL scores, OSA-18 total score, daytime sleepiness scales and ADHD rating scale between the resolved and residual groups [64].

In Isaiah A. et al., there were no associations identified between any of the polysomnographic parameters, including the AHI, and the behavioral outcomes. Although the parent-reported BRIEF MI score demonstrated the greatest improvement (Cohen *d* effect size = 0.5) in the early AT group, smaller improvements were identified for other parent reported behavioral outcomes (effect size for parent-reported Conners Global Index score was 0.3 in the early AT group). No statistically significant changes were identified for the teacher-reported BRIEF MI or the Conners Global Index scores (effect sizes for teacher-reported Conners Global Index and BRIEF MI scores were 0.1 for each outcome) [65].

#### Sleep Related Quality of Life after Surgery

Six articles, five RCTs [3,56,60,63,65] and one prospective cohort study [64] evaluated the effect of AT on the results of the questionnaires of sleep related quality of life (Table 4).

In Marcus et al.’s study, the symptoms of obstructive sleep apnea syndrome measured with the use of the Epworth Sleepiness Scale, the generic and disease-specific measures of quality of life and the PSQ-SRBD were assessed by means of the PedsQL and OSA-18, respectively. 

There was a more significant reduction in symptoms in the eAT group than in the WWSC group. At baseline, the PSQ-SRBD score and PedsQL score were as follows: 0.5 ± 0.2 and 76.5 ± 15.7 in WWSC group; and 0.5 ± 0.2 and 77.3 ± 15.3 in eAT group, with a change from baseline to 7 months after AT of −0.0 ± 0.2 (*p* < 0.001) and −0.3 ± 0.2 (*p* < 0.001) in two groups, respectively, for the PSQ-SRBD score and 0.9 ± 13.3 and 5.9 ± 13.6 in two groups, respectively, for the PedsQL score [3].

In Taylor et al.’s study, as in AHI outcomes, the regression analysis detected strong associations of improvement with positive changes in sleep parameters as measured by sleep questionnaires in the eAT group. The associations were weak (partial *rs* −0.15 to −0.30) and had small effect sizes (*f*^2^ 0.022–0.088) [56].

In Jeon et al.’s study, the mean KOSA-18s (Korean version of the obstructive sleep apnea-18 scores) at postoperative 1 month (32.2 ± 10.4) and at 6 months (32.5 ± 11.6) were significantly lower than the preoperative 1-day score (68.5 ± 19.9) (both *p* <0.01, paired *t-test*) [63].

In Rosen et al.’s study, the mean OSAS-18 score and ESS score at baseline (preoperative) were 52.8 ± 17.7 and 7.1 ± 4.7 and were 30.7 ± 13.8 at follow-up with a change from baseline to 7 months after AT of 21.9 ± 15.9 ((*p* < 0.001, Cohen’s d = 1.39 (trivial effect size)). The mean ESS at baseline (preoperative) was 7.1 ± 4.7, and it was 5.0 ± 4.4 at follow-up with a change from baseline to 7 months after AT of 22.0 ± 4.3 ((*p*< 0.001, Cohen’s d = 0.44 (medium effect size)) [60].

In Chun T. Au et al.’s study, the AT group had a significantly greater reduction in OSA-18 total score than the WW group (−17.3 ± 19.7 cf. −3.6 ± 14.1, *p* = 0.001) (Cohen d = −0.8 (trivial effect size)) [64]. 

In Isaiah A et al.’s study, the regression models revealed a positive association between the baseline PSQ-SRBD score and the parent-reported severity of behavioral impairment. Another mean (SD) decrease was identified for PSQ-SRBD scores in the early AT group vs. WWSC group (0.3 (0.2) vs. 0.0 (0.3)), resulting in a greater effect size (Cohen d = 1.5) [65].

## 4. Discussion

Contrasting data are reported on the effect of adenotonsillectomy for OSAS in children in behavior, cognitive function and quality of life improvements. Garetz et al. suggested that adenotonsillectomy is associated with improvements in these fields above, but new, large, randomized and controlled studies are needed to provide definitive evidence of the benefits of this surgical procedure [66].

In a meta-analysis of Yu et al., after 6–12 months of observation, significant improvements in attention-executive function and verbal ability were found in children with OSAS treated with AT compared to their baseline level. The Hedges’ g effect sizes of general intelligence, memory, attention-executive function and verbal ability compared to baseline level were medium (−0.37), medium (−0.36), trivial (−0.02) and medium (−0.45), respectively.

Moreover, the restoration of attention-executive function and memory was observed in children with OSAS after AT in comparison to healthy controls, and they say that rigorous randomized controlled trials should be conducted to obtain definitive conclusions [67].

Our review, which mainly includes RCT, reported better OSAS and sleep-related outcomes in tonsillectomies children, excluding the one that did not report PSG data. The six studies that included children with OSDB confirmed with PSG found that AHI (four studies) or ODI (two studies) or% TST EtCO2> 50 mmHg (2 studies) scores improved more in children receiving a tonsillectomy than in those who did not undergo surgery.

In six studies [3,56,59,63,64,65], the sleep-related quality of life after AT (PSQ-SRBD or ESS or OSA-18 or KOSA) improved with positive changes in sleep parameters (one study), or showed a significantly higher decrease in OSAS symptoms (one study) or was significantly improved than the baseline score (three studies). It is possible that the reductions in OSA symptoms and improvements observed in quality of life were affected by parental expectations [68].

Investigated changes in behavior and cognitive outcomes after AT were found to improve scores significantly post-AT in all studies. In only one study, comparing the AT group and control group, no differences reached significance at one year after AT and, in another one, did not show a significant improvement in all behavior and cognitive outcomes. In Paruthi et al.’s study [59], the hypercapnia did not correlate with changes on the cognitive and behavioral assessments at follow-up. In Isaiah et al.’s study [61], the results of this study demonstrated that the treatment related changes in behavioral outcomes were causally attributable solely to the changes in parent-reported OSDB severity.

There were no significant correlations between polysomnographic parameters and behavioral outcomes, and neither baseline hypercapnia nor change in EtCO2 levels predicted baseline or change in cognitive and behavioral parameters. We have noted that surgery resulted in more significant improvements in the BRIEF and in the scores on the Conners’ Rating Scale, measuring restlessness and impulsiveness and emotional lability, in the eAT group than in the WWSC group, and no significant difference between the two groups in the change from baseline to follow-up in our primary outcome was observed in terms of the attention and executive-function score of the NEPSY. The CBCL analysis confirmed an elevated prevalence of behavioral problems in children with OSAS at baseline. At follow-up, there was a highly significant improvement in Total Problems, Internalizing behaviors, Somatic Complaints and Thought Problems in children randomized to surgery than compared to WWSC. This review has shown a poor relation between neurocognitive/behavioral outcomes and polysomnographic parameters, even if improvements were found in the polysomnographic parameters and behaviors outcomes after AT. This may be because of the roles that affect many polysomnographic variables and other factors that affect behavior.

Of note, neither the PedsSQ nor the AHI predicted objectively measured attention or executive impairment at baseline or change after adenotonsillectomy.

The main practical implication of these results is that surgical candidacy for AT that is solely based on polysomnographic severity of SDB as measured by the AHI or other parameters is an unreliable predictor of behavioral outcomes. Conversely, standardized symptom-based questionnaires (e.g., PSQ-SRBD scale) should be considered as a useful adjunct for predicting behavioral outcomes in children undergoing AT.

Our systematic review, however, has several limitations, which are especially inherent in the nature of the evaluation tool performed for behavioral and cognitive performance assessment. Moreover, the samples enrolled in the indicated studies were not numerous, the study protocols were not adequate or there were no control groups. For this reason, further scientific evidence is needed to report data on the matter.

## 5. Conclusions

This review highlights the importance of future screening for behavioral symptoms and quality of life in children who present OSAS and compares behavioral symptoms and quality of life before and after AT as a predictive criterion. Currently, the PSG parameters provide clinicians with limited means to predict the improvement in neurobehavioral morbidity in OSAS. 

Despite the need for further research, this review suggests that AT treatment of OSAS can improve the behavioral symptoms of children with OSAS, possibly avoiding the need for psychopharmacological treatment. The clear improvement of patients after AT provides new suggestive evidence for a cause-and-effect relationship between sleep breathing disorders and various negative results on behavioral, cognitive and mental health.

Future studies should pay more attention to characterizing patient populations and should pay attention to using existing criteria such as severity of respiratory disorders and risk factor assessment. Moreover, attention needs to be paid to the evaluation of other comorbidities such as neuro-cognitive disorders in order to distinguish patients who need surgical treatment immediately and patients who can wait.

## Figures and Tables

**Table 1:** Studies characteristics.

Characteristic	RCTs (*n* = 7)	Prospective Cohort Studies (*n* = 4)	Total (*n* = 11)
Comparison watchful waiting group with AT group	7	1	8
Comparison OSAS group with the control group	/	2	2
Comparison, only OSAS group that underwent AT	1	1	2
PSG parameters reported	7	3	10
Sleep-related quality of life assessment	5	2	7
Cognitive and behavioural evalutation	7	4	11
Total participants, *n*	2186	590	2776

**Table 2:** Studies measured both at baseline and follow up.

Author	Year	Baseline Measures	Sample	Time of Follow-Up	Follow-Up Measures
(1) C.L. Marcus et al. [3]	2013	AHI NEPSY Conners’ Rating Scale-Revised BRIEF PSQ-SRBD PedsQL	464	7 months	AHI NEPSY Conners’ Rating Scale-Revised BRIEF PSQ-SRBD PedsQL
(2) H. G. Taylor et al. [56]	2016	AHI ODI PSQ-SRBD OSA-18 item mESS Verbal skills Nonverbal reasoning NEPSY-II Purdue Pegboard Test Developmental Test of Visual-Motor Integration WRAML2	453	7 months	Verbal skills Nonverbal reasoning NEPSY-II Word Generation Purdue Pegboard Test Developmental Test of Visual-Motor Integration WRAML2
(3) N. Hattiangadi Thomas et al. [57]	2017	AHI SpO2 nadir, % CBCL summary scores: Full CBCL (T-scores) Scale Scores Sleep item frequencies	380	7 months	CBCL summary scores: Full CBCL (T-scores) Scale Scores Sleep item frequencies
(4) K. Al-Zaabi et al. [58]	2018	AHI ODI CTRS-IA score CTRS-H score DST score COWAT score BSRT score TOL score RCPM score	37	3 months	AHI ODI CTRS-IA score CTRS-H score DST score COWAT score BSRT score TOL score RCPM score
(5) S. Paruthi et al. [59]	2015	AHI %TST EtCO2 > 50 mmHg NEPSY (Attention/Executive Function) Conners Rating Scale BRIEF	267	6 months	AHI %TST EtCO2 > 50 mmHg NEPSY (Attention/Executive Function) Conners Rating Scale BRIEF
(6) Y. J. Jeon et al. [63]	2016	Korean ADHD rating scale (K-ARS) Korean version of the obstructive sleep apnea-18 (KOSA-18) Preoperative attention-deficit domain Hyperactivity-impulsivity domain scores	148	postoperative 1 month 72 patients completed follow-up questionnaires at postoperative 6 months	Korean ADHD rating scale (K-ARS) Korean version of the obstructive sleep apnea-18 (KOSA-18) Preoperative attention-deficit domain Hyperactivity-impulsivity domain scores
(7) C. L. Rosen et al. [60]	2015	AHI ODI ETCO2 > 50 mm Hg NEPSY-A/E BRIEF Conners’ Rating Scale-Revised CBCL PedsQL (Child) PedsQL (Parent) OSAS-18 ESS, modified for children PSQ-SRBD	185	7 months	AHI ODI ETCO2 > 50 mm Hg NEPSY-A/E BRIEF Conners’ Rating Scale-Revised CBCL PedsQL (Child) PedsQL (Parent) OSAS-18 ESS, modified for children PSQ-SRBD
(8) J. E. Dillon et al. [61]	2007	Obstructive Apnea Index (OAI) Disruptive disorders: Attention-Deficit/Hyperactivity Disorder (ADHD) Oppositional Defiant Disorder (ODD) Anxiety/mood disorders: Disruptive Behavior Disorder Rating Scale (DBDRS) Children’s Global Assessment Scale (CGAS) Clinical Global Impressions (CGI) Depression factor score derived from the Children’ Psychiatric Rating Scale (CPRS Depression) Anxiety factor score derived from the children’s Psychiatric Rating Scale (CPRS Anxiety)	106	13 months	Obstructive Apnea Index (OAI) Disruptive disorders: Attention-Deficit/Hyperactivity Disorder (ADHD) Oppositional Defiant Disorder (ODD) Anxiety/mood disorders: Disruptive Behavior Disorder Rating Scale (DBDRS) Children’s Global Assessment Scale (CGAS) Clinical Global Impressions (CGI) Depression factor score derived from the Children’ Psychiatric Rating Scale (CPRS Depression) Anxiety factor score derived from the Children’ Psychiatric Rating Scale (CPRS Anxiety)
(9) R. D. Chervin et al. [62]	2006	AHI Mean sleep latency Cognitive attention Index Behavioural hyperactivity index	105	12 months	AHI Mean sleep latency Cognitive attention Index Behavioural hyperactivity index
(10) C. T. Au et al. [64]	2021	AHI ODI SpO2 Conners’ continuous performance test CBCL OSA-18 ESS Paediatric daytime sleepiness scale ADHD rating scale	114	6 months	AHI ODI SpO2 Conners’ continuous performance test CBCL OSA-18 ESS Paediatric daytime sleepiness scale ADHD rating scale
(11) Isaiah. A. et al. [65]	2020	AHI SpO2 Parent-reported Conners Global Index Teacher-reported Conners Global Index Parent-reported BRIEF Teacher-reported BRIEF Parent-reported CBCL internalizing problem subscale Parent-reported CBCL externalizing problem subscale Parent-reported CBCL total problems PSQ-SRBD	453	7 months	AHI SpO2 Parent-reported Conners Global Index Teacher-reported Conners Global Index Parent-reported BRIEF Teacher-reported BRIEF Parent-reported CBCL internalizing problem subscale Parent-reported CBCL externalizing problem subscale Parent-reported CBCL total problems PSQ-SRBD

Abbreviations: AHI = Apnea/Hypopnea Index; ODI = Oxigen Desaturation Index; NEPSY = Developmental Neuropsychological Assessment; BRIEF = Global Executive Composite T score; PSQ-SRBD = Pediatric Sleep Questionnaire sleep-related breathing disorder scale; PedsQL = Pediatric Quality of Life; ESS: Epworth Sleepiness Scale; CBCL = Child Behavior Checklist; CTRS = Conners’ Teacher Rating Scale; IA = inattention; H = hyperactivity; DST = Digit Span Test; COWAT = Controlled Oral Word Association Test; BSRT = Buschke Selective Reminding Test; TOL = Tower of London; RCPM = Raven’s Coloured Progressive Matrices; TST = Total Sleep Time; EtCO2 = End-tidal carbon dioxide.

**Table 3:** Baseline outcomes in WWSC group, control group or AT group.

Author/Year	Study Design	Country	Partecipant Design	Baseline Outcomes in WWSC or Control Group	Baseline Outcomes in AT Group
(1) C.L. Marcus et al. [3] 2013	randomized controlled trial	USA	Two OSAS groups: AT group vs. watchful waiting group	AHI: 4.5 events/h NEPSY: 101.1 ± 14.6 Conners’ Rating Scale-Revised: Caregiver rating: 52.6 ± 11.7 Teacher rating: 55.1 ± 12.8 BRIEF: Caregiver rating: 50.1 ± 11.5 Teacher rating: 56.4 ± 11.7 PSQ-SRBD: 0.5 ± 0.2 PedsQL: 76.5 ± 15.7	AHI: 4.8 events/h NEPSY: 101.5 ± 15.9 Conners’ Rating Scale-Revised: Caregiver rating: 52.5 ± 11.6 Teacher rating: 56.4 ± 14.4 BRIEF: Caregiver rating: 50.1 ± 11.2 Teacher rating: 57.2 ± 14.1 PSQ-SRBD: 0.5 ± 0.2 PedsQL: 77.3 ± 15.3
(2) H. G. Taylor et al. [56] 2016	randomized controlled trial	USA	Two OSAS groups: AT group vs. watchful waiting group	AHI: 4.51 (2.57–8.84) ODI: 4.71 (2.36–9.48) PSQ-SRBD: 0.50 (0.18) 18-item OSA: (18.83) mESS: 7.54 (5.15) Verbal skills: DAS-II Word Definitions 48.68 (8.15); DAS-II Verbal Similarities 49.10 (9.09); NEPSY Phonological Processing 8.49 (3.52); NEPSY Comprehension of Instructions 10.02 (2.84); NEPSY Speeded Naming 8.77 (3.30). Nonverbal reasoning: DAS-II Matrices 47.07 (7.83); DAS-II Sequential and Quantitative Reasoning 46.33 (8.67); DAS-II Pattern Construction 48.54 (7.62); -DAS-II Recall of Designs 48.46 (8.56). Attention and executive function: NEPSY Visual Attention 9.93 (2.89); NEPSY Auditory Attention and Response Set 10.04 (2.68); NEPSY Tower 10.52 (2.81); NEPSY-II Inhibition, Naming 8.59 (3.61); NEPSY-II Inhibition, Inhibition 8.08 (3.43); NEPSY-II Inhibition, Switching 8.02 (3.02); NEPSY-II Word Generation, Semantic Condition 10.60 (3.02); NEPSY-II Word Generation, Initial Letter Condition 8.81 (2.64). Perceptual–motor and visual-spatial skills: Purdue Pegboard Dominant Hand 0.03 (1.00); Purdue Pegboard Non-Dominant Hand −0.05 (1.09); Purdue Pegboard Both Hands 0.03 (0.97); Developmental Test of Visual-Motor Integration 95.09 (12.55); NEPSY Arrows 9.92 (2.77). Verbal learning and memory: WRAML2 Verbal Learning 10.04 (2.76); WRAML2 Verbal Learning Recall 10.26 (2.46); WRAML2 Verbal Learning Recognition 9.84 (2.87).	AHI: 4.79 (2.78–8.67) ODI: 4.97 (2.46–10.10) PSQ-SRBD: 0.49 (0.18) 18-item OSA: 53.12 (18.33) mESS: 7.08 (4.67) Verbal skills: DAS-II Word Definitions 49.78 (9.08); DAS-II Verbal Similarities 49.46 (7.69); NEPSY Phonological Processing 9.18 (3.24); NEPSY Comprehension of Instructions 10.25 (3.00); NEPSY Speeded Naming 8.99 (3.39). Nonverbal reasoning: DAS-II Matrices 47.96 (8.79); DAS-II Sequential and Quantitative Reasoning 45.93 (8.34); DAS-II Pattern Construction 48.97 (6.94); DAS-II Recall of Designs 48.21 (8.57). Attention and executive function: NEPSY Visual Attention 9.91 (2.87); NEPSY Auditory Attention and Response Set 9.99 (2.83); NEPSY Tower 10.70 (2.95); NEPSY-II Inhibition, Naming 8.84 (3.54); NEPSY-II Inhibition, Inhibition 7.83 (3.25); NEPSY-II Inhibition, Switching 8.01 (3.50); NEPSY-II Word Generation, Semantic Condition 10.29 (3.05); NEPSY-II Word Generation, Initial Letter Condition 8.91 (2.64). Perceptual–motor and visual-spatial skills: Purdue Pegboard Dominant Hand −0.03 (0.99); Purdue Pegboard Non-Dominant Hand 0.05 (0.90); Purdue Pegboard Both Hands −0.03 (1.02); Developmental Test of Visual-Motor Integration 94.33 (10.06); NEPSY Arrows 10.31 (2.80). Verbal learning and memory: WRAML2 Verbal Learning 10.00 (2.48); WRAML2 Verbal Learning Recall 10.00 (2.35); WRAML2 Verbal Learning Recognition 9.89 (3.03).
(3) *n*. Hattiangadi Thomas et al. [57] 2017	randomized controlled trial	USA	Two OSAS groups: AT group vs. watchful waiting group	AHI: 4.4 (2.5, 9.0) SpO2 nadir %: 90.0 (87, 92) CBCL summary scores: Full CBCL (T-scores): Total Problems: 53 Internalising: 52 Externalising: 51 Scale Scores: Anxious/Depressed: 51 Withdrawn/Depressed: 52 Somatic Complaints: 57 Social Problems: 53 Thought Problems: 54 Attention Problems: 53 Rule-Breaking Behavior: 53 Sleep item frequencies: Overtired Not true: 153 (79.7%) Somewhat/ sometimes true: 30 (15.6%) Very/often true: 9 (4.7%) Sleeps less Not true: 137 (71.4%) Somewhat/ sometimes true: 37 (19.3%) Very/often true: 18 (9.4%) Trouble sleeping Not true: 112 (58.3%) Somewhat/ sometimes true: 43 (22.4%) Very/often true: 37 (19.3%) Sleeps more Not true: 147 (76.6%) Somewhat/ sometimes true: 32 (16.7%) Very/often true: 13 (6.8%)Wets the bedNot true: 129 (67.2%) Somewhat/sometimes true: 39 (20.3%)Very/often true: 24 (12.5%)NightmaresNot true: 116 (60.4%) Somewhat/sometimes true: 64 (33.3%)Very/often true: 12 (6.3%)Talks/walks in sleepNot true: 128 (66.7%) Somewhat/sometimes true: 51 (26.56%)Very/often true: 13 (6.77%)	AHI: 4.8 (2.8, 8.8)SpO2 nadir %: 89.5 (86, 92)CBCL summary scores: Full CBCL (T-scores):Total Problems: 52 Internalising: 50Externalising: 51Scale Scores:Anxious/Depressed: 51Withdrawn/Depressed: 52Somatic Complaints: 53Social Problems:53Thought Problems: 54Attention Problems:53Rule-Breaking Behavior: 52Sleep item frequencies:OvertiredNot true: 129 (70.1%) Somewhat/sometimes true: 41 (22.3%)Very/often true: 14 (7.6%)Sleeps lessNot true 139 (75.5%) Somewhat/sometimes true: 34 (18.5%)Very/often true: 11 (6.0%)Trouble sleepingNot true: 108 (58.7%) Somewhat/sometimes true: 48 (26.1%)Very/often true: 28 (15.2%)Sleeps moreNot true: 144 (78.3%) Somewhat/ sometimes true: 35 (19.0%) Very/often true: 5 (2.7%) Wets the bed Not true: 130 (70.7%) Somewhat/ sometimes true: 30 (16.3%) Very/often true: 24 (13.0%) Nightmares Not true: 118 (64.1%) Somewhat/ sometimes true: 60 (32.6%) Very/often true: 6 (3.3%) Talks/walks in sleep Not true: 121 (65.8%) Somewhat/ sometimes true: 54 (29.4%) Very/often true: 9 (4.9%)
(4) K. Al-Zaabi et al. [58] 2018	prospective cohort study	Oman	One OSAS group: AT group	n/a	AHI: 5.37 ± 7.17 ODI: 5.19 ± 8.14 CTRS-IA score: 18.76 ± 4.79 CTRS-H score: 19.92 ± 6.72 DST score: 6.83 ± 2.69 COWAT score: 6.07 ± 5.45 BSRT score: 18.65 ± 5.72 TOL score: 11.46 ± 4.74 RCPM score: 18.03 ± 7.47
(5) S. Paruthi et al. [59] 2015	randomized controlled trial	USA	Two OSAS groups: AT group vs. watchful waiting group	AHI: 6.2 %TST EtCO2 > 50 mmHg: 8.3 NEPSY (Attention/Executive Function): n/a Conners Rating Scale: n/a BRIEF: n/a	AHI: 6.8 %TST EtCO2 > 50 mmHg: 12.9 NEPSY (Attention/Executive Function): n/a Conners Rating Scale: n/a BRIEF: n/a
(6) Y. J. Jeon et al. [63] 2016	randomized controlled trial	KOREA	One OSAS group: AT group	n/a	K-ARS: 12.5 ± 9.7 (KOSA-18): 32.2 ± 10.4 Preoperative attention-deficit domain: 6.2 ± 5.3 Hyperactivity-impulsivity domain scores: 6.2 ± 5.1
(7) C. L. Rosen et al. [60] 2015	randomized controlled trial	USA	One OSAS group: AT group		AHI: 4.8 (6.4); 1.2–27.7 ODI: 4.9 (8); 0.0–32.8 ETCO2 > 50 mm Hg: 2.0 (13.5); 0–86.8 NEPSY-A/E: 30.9 ± 6.2 BRIEF: 49.5 6 10.8 46.2 ± 11.3 Conners’ Rating Scale Revised: 52.1 ± 11.4 CBCL: 52.1 ± 10.9 PedsQL (Child): 68.8 ± 15.4 PedsQL (Parent): 78.8 ± 15.4 OSAS-18: 52.8 ± 17.7 ESS, modified for children: 7.1 ± 4.7 Pediatric Sleep Questionnaire (PSQ): 0.49 ± 0.18; 0.05–0.95
(8) J. E. Dillon et al. [61] 2007	prospective cohort study	USA	Tree goups: AT group with OSAS vs. AT group without OSAS vs. control group	Obtructive Apnea Index (OAI):0.2 (0.4) Disruptive disorders: 3 (11.1) ADHD: 2 (7.4) ODD (oppositional defiant disorder): 1 (3.7) Anxiety/mood disorders: 2 (7.4) Disruptive Behavior Disorder Rating Scale (DBDRS): DBDRS-IA: 4.6 (1.3) DBDRS-HI: 5.9 (1.1) DBDRS-combined: 10.4 (2.1) DBDRS-ODD: 1.4 (0.8) Children’s Global Assessment Scale (CGAS): 76.0 (2.6) Clinical Global Impressions (CGI): 1.7 (0.2) Depression factor score derived from theChildren’s Psychiatric Rating Scale (CPRS Depression): −0.178 (0.21) Anxiety factor score derived from the children’s Psychiatric Rating Scale (CPRS Anxiety): −0.047 (0.21)	Obtructive Apnea Index (OAI): 5.6 (8.0) Disruptive disorders: 29 (36.7) ADHD: 22 (27.8) ODD (oppositional defiant disorder): 14 (17.7) Anxiety/mood disorders: 14 (17.7) Disruptive Behavior Disorder Rating Scale (DBDRS): DBDRS-IA: 7.5 (1.2) DBDRS-HI: 6.4 (0.6) DBDRS-combined: 13.9 (1.1) DBDRS-ODD: 4.8 (0.4) Children’s Global Assessment Scale (CGAS): 68.2 (1.4) Clinical Global Impressions (CGI): 2.5 (0.1) Depression factor score derived from the children’s Psychiatric Rating Scale (CPRS Depression): 0.186 (0.11) Anxiety factor score derived from the children’s Psychiatric Rating Scale (CPRS Anxiety): 0.173 (0.11)
(9) R. D. Chervin et al. [62] 2006	prospective cohort study	USA	Two groups: AT group vs. control group	AHI: 1.2 ± 1.9 Mean sleep latency: n/a Cognitive attention Index: n/a Behavioural hyperactivity index: n/a	AHI: 7.3 ± 12.5 Mean sleep latency: n/a Cognitive attention Index: n/a Behavioral hyperactivity index: n/a
(10) Chun T. Au et al. [64] 2021	prospective cohort study	CHINA (HONG KONG)	Two OSAS groups: AT group vs. watchful waiting group	AHI: 3.3 ± 1.6 ODI: 2.3 ± 2.2 SpO_2_: 91 ± 4 CBCL: Total problems T score: 59 ± 9 Conners’ continuous performance test: Inattentivenes (Hit reaction time): 51 ± 12 Impulsivity (Hit reaction time: 51 ± 12 OSA-18: 56 ± 16 ESS: 6 ± 4 Paediatric daytime sleepiness scale: 14 ± 5 ADHD rating: Total: 20 ± 9	AHI: 3.5 ± 1.6 ODI: 1.8 ± 1.7 SpO_2_: 93 ± 2 CBCL: Total problems T score: 59 ± 9 51 ±15 1.5 ± 9.7 Conners’ continuous performance test: Inattentivenes (Hit reaction time): 51 ± 15 Impulsivity (Hit reaction time: 51 ± 15 OSA-18: 63 ± 17 ESS: 7 ±4 Paediatric daytime sleepiness scale: 16 ± 4 ADHD rating: Total: 21 ± 10
(11) I. Arnal et al. [65] 2020	randomized controlled trial	USA	Two OSAS groups: AT group vs. watchful waiting group	(Mean (SD) between groups) AHI n/a SpO2 n/a Parent-reported Conners Global Index: 52.5 (11.7) Teacher-reported Conners Global Index: 50.3 (11.4) Parent-reported BRIEF: 59.1 (16.8) Teacher-reported BRIEF: 59.6 (20.8) Parent-reported CBCL internalizing problem subscale: 52.0 (11.6) Parent-reported CBCL externalizing problem subscale: 51.6 (11.3) Parent-reported CBCL total problems: 53.1 (11.0) PSQ-SRBD: n/a	

AHI = Apnea/Hypopnea Index; ODI = Oxigen Desaturation Index; NEPSY = Developmental Neuropsychological Assessment; BRIEF = Global Executive Composite T score; PSQ-SRBD = Pediatric Sleep Questionnaire sleep-related breathing disorder scale; PedsQL = Pediatric Quality of Life; ESS = Epworth Sleepiness Scale; CBCL = Child Behavior Checklist; CTRS = Conners’ Teacher Rating Scale; IA = inattention; H = hyperactivity;DST = Digit Span Test; COWAT = Controlled Oral Word Association Test; BSRT = Buschke Selective Reminding Test; TOL = Tower of London; RCPM = Raven’s Coloured Progressive Matrices; TST = Total Sleep Time; EtCO2 = End-tidal carbon dioxide.

**Table 4:** Follow-up outcomes in WWSC group, control group or AT group and change from baseline to follow-up between groups.

Author/Year	Study Design	Country	Partecipant Design	Outcomes at Follow-Up in WWSC or Control Group	Outcomes at Follow-Up in AT Group	Change from Baseline to Follow-Up between Groups (*p* Value)
(1) C.L. Marcus et al. [3] 2013	randomized controlled trial	USA	Two OSAS groups: AT group vs. watchful waiting group	AHI: reduced by −1.6 events/h NEPSY: Average scores increased 5.1 ± 13.4 Conners’ Rating Scale-Revised: Caregiver rating: −0.2 ± 9.4 Teacher rating: −1.5 ± 10.7 BRIEF: Caregiver rating: 0.4 ± 8.8 Teacher rating: −1.0 ± 11.2 PSQ-SRBD: −0.0 ± 0.2 PedsQL: 0.9 ± 13.3	AHI: reduced by −3.5 events/h NEPSY: 7.1 ± 13.9 Conners’ Rating Scale-Revised: Caregiver rating: −2.9 ± 9.9 Teacher rating: −4.9 ± 12.9 BRIEF: Caregiver rating: −3.3 ± 8.5 Teacher rating: −3.1 ± 12.6 PSQ-SRBD: −0.3 ± 0.2 PedsQL: 5.9 ± 13.6	AHI: (*p* < 0.001) NEPSY:Average scores increased (*p* = 0.16) Conners’ Rating Scale-Revised: Caregiver rating: Average scores improves significantly: (*p* = 0.01) Teacher rating: Average scores improves significantly: (*p* = 0.04) BRIEF: Average scores improves significantly only for Caregiver rating: (*p* < 0.001) PSQ-SRBD: Average scores improves significantly:(*p* < 0.001) PedsQL: Average scores improves significantly: (*p* < 0.001)
(2) H. G. Taylor et al. [56] 2016	randomized controlled trial	USA	Two OSAS groups: AT group vs. watchful waiting group	AHI: n/a ODI: n/a PSQ-SRBD: n/a 18-item OSA: n/a mESS: n/a Verbal skills: DAS-II Word Definitions 49.33 (8.25); DAS-II Verbal Similarities 50.22 (8.77); NEPSY Phonological Processing 8.98 (3.14); NEPSY Comprehension of Instructions 10.18 (2.91); NEPSY Speeded Naming 9.43 (3.40). Nonverbal reasoning: DAS-II Matrices 47.84 (9.69); DAS-II Sequential and Quantitative Reasoning 46.71 (8.96); DAS-II Pattern Construction 49.92 (7.54); DAS-II Recall of Designs 49.67 (8.25). Attention and executive function: NEPSY Visual Attention 10.36 (2.88); NEPSY Auditory Attention and Response Set 10.68 (2.90); NEPSY Tower 11.28 (2.71); NEPSY-II Inhibition, Naming 8.90 (3.65); NEPSY-II Inhibition, Inhibition 8.76 (3.44); NEPSY-II Inhibition, Switching 8.33 (3.25); NEPSY-II Word Generation, Semantic Condition 10.77 (3.08); NEPSY-II Word Generation, Initial Letter Condition 9.24 (3.17). Perceptual–motor and visual-spatial skills: Purdue Pegboard Dominant Hand 0.15 (1.05); Purdue Pegboard Non-Dominant Hand 0.15 (1.14); Purdue Pegboard Both Hands −0.04 (0.80); Developmental Test of Visual-Motor Integration 93.91 (10.93); NEPSY Arrows 10.28 (2.67). Verbal learning and memory: WRAML2 Verbal Learning 10.75 (2.77); WRAML2 Verbal Learning Recall 10.23 (2.65); WRAML2 Verbal Learning Recognition 10.28 (2.46).	AHI: n/a ODI: n/a PSQ-SRBD: n/a 18-item OSA: n/a mESS: n/a Verbal skills: DAS-II Word Definitions 50.41 (8.35); DAS-II Verbal Similarities 50.30 (8.72); NEPSY Phonological Processing 9.39 (3.52); NEPSY Comprehension of Instructions 10.45 (3.07); NEPSY Speeded Naming 9.64 (3.11). Nonverbal reasoning: DAS-II Matrices 49.88 (8.78); DAS-II Sequential and Quantitative Reasoning 48.03 (8.67); DAS-II Pattern Construction 49.76 (6.98); DAS-II Recall of Designs 49.49 (8.31). Attention and executive function: NEPSY Visual Attention 10.96 (3.03); NEPSY Auditory Attention and Response Set 10.81 (2.62); NEPSY Tower 11.53 (2.81); NEPSY-II Inhibition, Naming 9.30 (3.72); NEPSY-II Inhibition, Inhibition 9.11 (3.42); NEPSY-II Inhibition, Switching 9.21 (3.81); NEPSY-II Word Generation, Semantic Condition 10.51 (3.07); NEPSY-II Word Generation, Initial Letter Condition 9.16 (2.96). Perceptual–motor and visual-spatial skills: Purdue Pegboard Dominant Hand 0.27 (0.96); Purdue Pegboard Non-Dominant Hand 0.18 (1.10); Purdue Pegboard Both Hands 0.10 (0.81); Developmental Test of Visual-Motor Integration 93.94 (11.34); NEPSY Arrows 10.46 (2.72). Verbal learning and memory: WRAML2 Verbal Learning 10.71 (2.86); WRAML2 Verbal Learning Recall 10.22 (2.72); WRAML2 Verbal Learning Recognition 10.40 (3.01).	Verbal skills: not significant change (*p* = 0.942; *p* = 0.557; *p* = 0.443; *p* = 0.803; *p* = 0.773) Nonverbal reasoning: significant change only for DAS-II Sequential and Quantitative Reasoning (*p* = 0.040) Attention and executive function: not significant change (*p* = 0.061; *p* = 0.353; *p* = 0.960; *p* = 0.739; *p* = 0.072; *p* = 0.201; *p* = 0.797; *p* = 0.580) Perceptual–motor and visual-spatial skills: significant change only for Purdue Pegboard Both Hands (*p* = 0.030) Verbal learning and memory: not significant change (*p* = 0.935; *p* = 0.240; *p* = 0.992)
(3) N. Hattiangadi Thomas et al. [57] 2017	randomized controlled trial	USA	Two OSAS groups: AT group vs. watchful waiting group	AHI: n/a SpO2 nadir%: n/a CBCL summary scores: Full CBCL (T-scores): Total Problems: −1 Internalizing: −1 Externalising: 0 Scale Scores: Anxious/Depressed: 0 Withdrawn/Depressed: 0 Somatic Complaints: 0 Social Problems: 0 Thought Problems: 0 Attention Problems: 0 Rule-Breaking Behavior: 0 Sleep item frequencies: Overtired Not true: 156 (81.3%) Somewhat/ sometimes true: 29 (15.1%) Very/often true: 7 (3.7%) Sleeps less Not true: 153 (79.7%) Somewhat/ sometimes true: 26 (13.5%) Very/often true: 13 (6.8%) Trouble sleeping Not true: 135 (70.3%) Somewhat/ sometimes true: 37 (19.3%) Very/often true: 20 (10.4%) Sleeps more Not true: 156 (81.3%) Somewhat/ sometimes true: 24 (12.5%) Very/often true: 12 (6.3%) Wets the bed Not true: 140 (72.9%) Somewhat/ sometimes true: 38 (19.8%) Very/often true: 14 (7.3%) Nightmares Not true: 121 (63.0%) Somewhat/ sometimes true: 66 (34.4%) Very/often true: 5 (2.6%) Talks/walks in sleep Not true: 129 (67.19%) Somewhat/ sometimes true: 52 (27.08%) Very/often true: 11 (5.73%)	AHI: n/a SpO2 nadir%: n/a CBCL summary scores: Full CBCL (T-scores): Total Problems: −4 Internalizing: −3 Externalizing−2 Scale Scores: Anxious/Depressed: 0 Withdrawn/Depressed: 0 Somatic Complaints: 0 Social Problems: 0 Thought Problems: −1 Attention Problems: 0 Rule-Breaking Behavior: 0 Sleep item frequencies: Overtired Not true: 162 (88.0%) Somewhat/ sometimes true: 20 (10.9%) Very/often true: 2 (1.1%) Sleeps less Not true: 160 (87.0%) Somewhat/ sometimes true: 22 (12.0%) Very/often true: 2 (1.1%) Trouble sleeping Not true: 152 (82.6%) Somewhat/ sometimes true: 25 (13.6%) Very/often true: 7 (3.8%) Sleeps more Not true: 166 (90.2%) Somewhat/ sometimes true: 15 (8.2%) Very/often true: 3 (1.6%) Wets the bed Not true: 148 (80.4%) Somewhat/ sometimes true: 20 (10.9%) Very/often true: 16 (8.7%) Nightmares Not true: 133 (72.3%) Somewhat/ sometimes true: 46 (25.0%) Very/often true: 5 (2.7%) Talks/walks in sleep Not true: 146 (79.4%) Somewhat/ sometimes true: 35 (19.0%) Very/often true: 3 (1.6%)	CBCL summary scores: Full CBCL (T-scores): significant changes for Total Problems (*p* < 0.001) and Internalizing: (*p* = 0.04) Scale Score: significant changes only for Somatic Complaints (*p* = 0.01) and Thought Problems (*p* = 0.01) Sleep item frequencies: significant changes only for “Overtired item” (*p* = 0.01) and “Talks/walks in sleep item”(*p* = 0.01)
(4) K. Al-Zaabi et al. [58] 2018	prospective cohort study	Oman	One OSAS group: AT group	n/a	AHI: 2.36 ±4.88 ODI: 1.14 ± 2.87 CTRS-IA score: 14.86 ± 3.65 CTRS-H score: 15.84 ± 4.13 DST score: 9.70 ± 4.51 COWAT score: 11.67 ± 8.39 BSRT score: 25.65 ± 5.49 TOL score: 17.43 ± 5.19 RCPM score: 23.97 ± 7.19	AHI: improves significantly: (*p* < 0.01) ODI: improves significantly: (*p* < 0.01) CTRS-IA score: improves significantly: (*p* < 0.01) CTRS-H score: improves significantly: (*p* < 0.01) DST score: improves significantly: (*p* < 0.01) COWAT score: improves significantly: (*p* < 0.01) BSRT score: improves significantly: (*p* < 0.01) TOL score: improves significantly: (*p* < 0.01) RCPM score: improves significantly: (*p* < 0.01)
(5) S. Paruthi et al. [59] 2015	randomized controlled trial	USA	Two OSAS groups: AT group vs. watchful waiting group	AHI:5.1 %TST EtCO2 > 50 mmHg:7.5 NEPSY (Attention/Executive Function): n/a Conners Rating Scale: n/a BRIEF: n/a	AHI:1.5 %TST EtCO2 > 50 mmHg: 9.9 NEPSY (Attention/Executive Function): n/a Conners Rating Scale: n/a BRIEF: n/a	AHI: improvement in the eAT group relative to the WWSC group was about twice as high as the improvement in EtCO2 between groups (*p* < 0.0001,Cohen’d effect size of 0.61). %TST EtCO2 > 50 mmHg: showed significantly more improvement in the AT group compared to the WWSC group (*p* = 0.010, Cohen d effect size of 0.32). TST with EtCO2> 50 mmHg did not correlate with changes on the cognitive and behavioral assessments (NEPSY, Conners Rating Scale and BRIEF ) at follow-up (r = 0.0.09 to 0.0.012, all *p* > 0.15).
(6) Y. J. Jeon et al. [63] 2016	randomized controlled trial	KOREA	One OSAS group: AT group	n/a	K-ARS at 1 month: 7.0 ± 6.4 K-ARS at 6 mo: 8.4 ± 7.7 (KOSA-18) at 1 mo: 31.9 ± 8.3 (KOSA-18) at 6 mo: 32.5 ± 11.6 attention deficit domain at 1 mo: 3.1 ± 3.2 hyperactivity-impulsivity domain scores at 1 mo: 3.9 ± 3.6 attention deficit domain at 6 mo: 4.1 ± 3.8 hyperactivity-impulsivity domain scores at 6 mo: 4.7 ± 4.7	K-ARS at 1 month: improves significantly: (*p* < 0.01) K-ARS at 6 mo: improves significantly: (*p* < 0.01) (KOSA-18) at 1 mo: improves significantly: (*p* < 0.01) (KOSA-18) at 6 mo: improves significantly: (*p* < 0.01) attention deficit domain at 1 mo: decresed significantly: (*p* < 0.01) hyperactivity-impulsivity domain scores at 1 mo: decresed significantly: (*p* < 0.01) attention deficit domain at 6 mo: decresed significantly: (*p* < 0.01) hyperactivity-impulsivity domain scores at 6 mo: decresed significantly: (*p* < 0.01)
(7) C. L. Rosen et al. [60] 2015	randomized controlled trial	USA	One OSAS group: AT group	n/a	AHI: reduced by −3.5 (6.1), ODI: n/a ETCO2 > 50 mm Hg: n/a NEPSY-A/E: 33.5 ± 5.9 BRIEF: 46.2 ± 11.3 Conners’ Rating Scale Revised: 49.2 ± 10.8 CBCL: 48.2 ± 12.0 PedsQL (Child): 72.3 ± 15.2 PedsQL (Parent): 84.5 ± 14.9 OSAS-18: 30.7 ± 13.8 ESS, modified for children: 5.0 ± 4.4 Pediatric Sleep Questionnaire (PSQ): n/a	NEPSY-A/E: significant change 2.7 ± 5.2, (*p* < 001, effect size −0.43) BRIEF: significant change −3.3 ± 8.3 (*p* < 001, effect size 0.30) Conners’ Rating Scale Revised: significant change −2.9 ± 9.9 (*p* < 001, effect size 0.26) CBCL: significant change −3.9 ± 8.0 (*p* < 001, effect size 0.34) PedsQL (Child): no significant change 3.6 ± 17.2 (*p* < 0.06, effect size −0.23) PedsQL (Parent): significant change 5.7 ± 14.6 (*p* < 0.06, effect size −0.37) OSAS-18: significant change −21.9 ± 15.9 (*p* < 001, effect size 1.39) ESS, modified for children: −2.0 ± 4.3 (*p* < 001, effect size 0.44)
(8) J. E. Dillon et al. [61] 2007	prospective cohort study	USA	Two goups: AT group with OSAS vs. control group	Obtructive Apnea Index (OAI): 0.2 Disruptive disorders: 2 ADHD: 2 Oppositional Defiant Disorder (ODD): 0 Anxiety/mood disorders: 0 Disruptive Behavior Disorder Rating Scale (DBDRS): DBDRS-IA: 4.1 (1.3) DBDRS-HI: 4.4 (1.1) DBDRS-combined: 8.5 (2.1) DBDRS-ODD: 1.3 (0.8) Children’s Global Assessment Scale (CGAS): 77.5 (2.6) Clinical Global Impressions (CGI): 1.5 (0.2) Depression factor score derived from the children’s Psychiatric Rating Scale (CPRS Depression): −0.448 (0.21) Anxiety factor score derived from the children’s Psychiatric Rating Scale (CPRS Anxiety): −0.286 (0.21)	Obtructive Apnea Index (OAI): 0.2 Disruptive disorders: 18 ADHD: 16 Oppositional Defiant Disorder (ODD): 7 Anxiety/mood disorders: 8 Disruptive Behavior Disorder Rating Scale (DBDRS): DBDRS-IA: 5.9 (0.7) DBDRS-HI: 4.7 (0.6) DBDRS-combined: 10.6 (1.1) DBDRS-ODD: 3.4 (0.4) Children’s Global Assessment Scale (CGAS): 72.3 (1.4) Clinical Global Impressions (CGI): 2.0 (0.1) Depression factor score derived from the children’s Psychiatric Rating Scale (CPRS Depression): −0.001 (0.11) Anxiety factor score derived from the children’s Psychiatric Rating Scale (CPRS Anxiety): −0.075 (0.11)	Disruptive disorders: significant change *p* = 0.015 (Fisher’s exact test) ADHD: no significant change: *p* = 0.033 (Fisher’s exact test). Oppositional Defiant Disorder (ODD): significant change *p* = 0.108 (Fisher’s exact test). Anxiety/mood disorders: no significant change: *p* = 0.132 (McNemar’s test) (DBDRS): DBDRS-IA: no significant main effects (*p* = 0.071) DBDRS-HI: significant main effects (*p* = 0.002) DBDRS-combined: significant main effects (*p* = 0.007) DBDRS-ODD: (*p* = 0.040) (CGAS): significant main effects (*p* = 0.035) (CGI): significant main effects (*p* = 0.003) (CPRS Anxiety): significant main effects (*p* = 0.026)
(9) R. D. Chervin et al. [62] 2006	prospective cohort study	USA	Two groups: AT group vs. control group	AHI: 1.2 ± 1.8 Mean sleep latency: n/a Cognitive attention Index: n/a Behavioral hyperactivity index: n/a	AHI: 1.1 ± 1.1 Mean sleep latency: n/a Cognitive attention Index: n/a Behavioural hyperactivity index: n/a	AHI: no significant change: *p* = 0.91 Mean sleep latency: no significant change: *p* = 0.745 Cognitive attention Index: no significant change: *p* = 0.133 Behavioural hyperactivity index: no significant change: *p* = 0.056
(10) Chun T. Au et al. [64] 2021	prospective cohort study	CINA (HONG KONG)	Two OSAS groups: AT group vs. watchful waiting group	AHI: Change from baseline 0.2 ± 4.4 ODI: Change from baseline 0.5 ±3.9 SpO_2_: Change from baseline 0.8 ± 4.6 CBCL: Total problems T score: Change from baseline −2.9 ±8.0 Conners’ continuous performance test: Inattentivenes (Hit reaction time): Change from baseline −2.0 ±7.9 OSA-18: Change from baseline −3.6 ±14.1 ESS: −0.7 ±2.9 Paediatric daytime sleepiness scale: Change from baseline −1.0 ± 4.8 ADHD rating: Total: Change from baseline −1.8 ± 8.6	AHI: Change from baseline −1.1 ±2.3 ODI: Change from baseline −0.4 ±2.0 SpO_2_: Change from baseline −0.1 ± 3.9 CBCL: Total problems T score: Change from baseline −4.4 ± 8.9 Conners’ continuous performance test: Inattentivenes (Hit reaction time): Change from baseline 1.5 ± 9.7 OSA-18: Change from baseline −17.3 ±19.7 ESS: Change from baseline −1.0 ± 5.0 Paediatric daytime sleepiness scale: Change from baseline −1.6 ± 5.4 ADHD rating: Total: Change from baseline −3.5 ±9.7	AHI: no significant change: *p* = 0.12 ODI: no significant change: *p* = 0.22 SpO_2_: no significant change: *p* = 0.39 CBCL: Total problems T score: no significant change: *p* = 0.45 Conners’ continuous performance test: Inattentivenes (Hit reaction time): no significant change: *p* = 0.097 OSA-18: significant change: *p* = 0.001 ESS: no significant change: *p* = 0.75 Paediatric daytime sleepiness scale: no significant change: *p* = 0.61 ADHD rating: Total: no significant change: *p* = 0.43
(11) I. Arnal et al. [65] 2020	randomized controlled trial	USA	Two OSAS groups: AT group vs. watchful waiting group	AHI n/a SpO2 n/a Parent-reported Conners Global Index: Change from baseline –0.3 (9.4) Teacher-reported Conners Global Index: Change from baseline –1.6 (20.4) Parent-reported BRIEF: Change from baseline 0.3 (9.0) Teacher-reported BRIEF: Change from baseline 0.0 (23.5) Parent-reported CBCL Internalizing problem subscale: Change from baseline –0.7 (9.5) Parent-reported CBCL externalizing problem subscale: Change from baseline –1.3 (7.9) Parent-reported CBCL total problems: Change from baseline 1.1 (8.3) PSQ-SRBD: n/a	AHI n/a SpO2 n/a Parent-reported Conners Global Index: Change from baseline −2.8 (10.2) Teacher-reported Conners Global Index: Change from baseline −3.5 (19.6) Parent-reported BRIEF: Change from baseline –3.7 (8.1) Teacher-reported BRIEF: Change from baseline –1.0 (22.8) Parent-reported CBCL internalizing problem subscale: Change from baseline –4.0 (11.3) Parent-reported CBCL externalizing problem subscale: Change from baseline –2.3 (8.5) Parent-reported CBCL total problems: Change from baseline –4.2 (8.7) PSQ-SRBD: n/a	AHI: The mean (SD) decrease in the AHI score was 5.2 (6.3) for AT group and was 0.7 (6.4) for WWSC group (Cohen d = 0.6) Parent-reported Conners Global Index: Cohen d effect size = 0.30 Teacher-reported Conners Global Index: Small effect size (Cohen d = 0.01) Parent-reported BRIEF: Medium effect size (Cohen d = 0.50) Teacher-reported BRIEF: Small effect size (Cohen d = 0.01) Parent-reported CBCL internalizing problem subscale: Small effect size (Cohen d = 0.39) Parent-reported CBCL externalizing problem subscale: Small effect size (Cohen d = 0.14) Parent-reported CBCL total problems: Small effect size (Cohen d = 0.37) PSQ-SRBD: A greater mean (SD) decrease was identified for PSQ-SRBD scores in the AT group vs. WWSC group (0.3 (0.2) vs. 0.0 (0.3)), resulting in a greater effect size (Cohen d = 1.5)

AHI = Apnea/Hypopnea Index; ODI = Oxigen Desaturation Index; NEPSY = Developmental Neuropsychological Assessment; BRIEF = Global Executive Composite T score; PSQ-SRBD = Pediatric Sleep Questionnaire sleep-related breathing disorder scale; PedsQL = Pediatric Quality of Life; ESS = Epworth Sleepiness Scale; CBCL = Child Behavior Checklist; CTRS = Conners’ Teacher Rating Scale; IA = inattention; H = hyperactivity;DST = Digit Span Test; COWAT = Controlled Oral Word Association Test; BSRT = Buschke Selective Reminding Test; TOL = Tower of London; RCPM = Raven’s Coloured Progressive Matrices; TST = Total Sleep Time; EtCO2 = End-tidal carbon dioxide.

## Data Availability

Data are available upon request.
